# Chemical and Non-Chemical Options for Managing Twospotted Spider Mite, Western Tarnished Plant Bug and Other Arthropod Pests in Strawberries

**DOI:** 10.3390/insects9040156

**Published:** 2018-11-01

**Authors:** Surendra K. Dara, David Peck, Dave Murray

**Affiliations:** 1 University of California Cooperative Extension, Division of Agriculture and Natural Resources, San Luis Obispo, CA 93401, USA; 2 Manzanita Berry Farms, Santa Maria, CA 93456, USA; dpeck83@gmail.com; 3 Sundance Berry Farms, Watsonville, CA 95076, USA; drm2472@gmail.com

**Keywords:** *Frankliniella occidentalis*, *Lygus hesperus*, *Trialeurodes vaporariorum*, *Tetranychus urticae*, entomopathogenic fungi, integrated pest management

## Abstract

California strawberries have two major arthropod pests—the twospotted spider mite, *Tetranychus urticae* and the western tarnished plant bug, *Lygus hesperus*, which result in significant losses to the yield and quality of marketable berries. Other important insect pests that are frequently seen in strawberry include the greenhouse whitefly, *Trialeurodes vaporariorum* and the western flower thrips, *Frankliniella occidentalis* that cause varying levels of damage depending on the level of infestation. Chemical pesticides play a major role in managing these pests but not without the associated risk of pesticide resistance and environmental safety. Two field studies were conducted in commercial strawberry fields in Santa Maria, one of the strawberry growing areas in California Central Coast, to determine the efficacy of chemical, botanical and microbial pesticides in the integrated pest management (IPM) of strawberry. Chemical, botanical and microbial pesticides were evaluated against *T. urticae* in a small plot study in 2013 and against *L. hesperus* and other insect pests in a large plot study in 2015 in commercial strawberry fields. Bug vacuums were also used in the 2015 study. Results demonstrated that non-chemical alternatives can play an important role in strawberry IPM.

## 1. Introduction

Strawberry is the fourth most valuable commodity in California with a crop value of $3.1 billion [1]. The twospotted spider mite, *Tetranychus urticae* Koch (Acari: Tetranychidae), and the western tarnished plant bug (commonly referred to as lygus bug), *Lygus hesperus* Knight (Hemiptera: Miridae), are two major arthropod pests that cause significant losses to the yield and quality of marketable berries every season [2]. Other important insect pests that can cause significant damage when populations are high include the greenhouse whitefly, *Trialeurodes vaporariorum* (Westwood) (Hemiptera: Aleyrodidae), and the western flower thrips, *Frankliniella occidentalis* (Pergande) (Thysanoptera: Thripidae). *Tetranychus urticae* is generally found on the underside of the leaves scraping the epidermis and feeding on the plant juices. Mite damage reduces plant vigor and thus fruit yields. Severe mite infestations can also lead to plant death. Nymphs and adults of *L. hesperus* feed on developing seeds and surrounding areas on the fruit using their piercing and sucking mouthparts. As the fruit develops, damaged tissues result in uneven growth and cause fruit deformity. Deformed berries are not marketable and contribute to yield losses. Populations of *T. vaporaiorum* and *F. occidentalis* are usually kept below threshold levels with treatments targeted towards other pests but can occasionally build up to damaging levels. When *T. vaporariorum* numbers are high, honeydew secretion and resulting sooty mold development on the foliage can affect photosynthesis and reduce the quality of fruit. They can also transmit viruses resulting in the viral decline of strawberry [3]. High *F. occidentalis* infestations cause discoloration known as bronzing on the fruit [2]. Biological control of *T. urticae* is a popular practice in California strawberry where different species of commercially-produced predatory mites are periodically released [2]. Management of *L. hesperus* continues to be a major challenge for California strawberry growers. Although earlier studies showed limited or varying levels of control of *L. hesperus* from vacuums, their use is becoming popular especially on some large farms [2,4,5,6]. Strawberry is one of the crops where large quantities of pesticides are used and *T. urticae* and *L. hesperus* are important targets for miticide and insecticide applications [7].

Excessive use of chemicals leads to pesticide resistance, making it more challenging to manage pests that are already difficult to control [8,9,10]. Although several botanical and microbial pesticides are registered against *L. hesperus*, *T. urticae* and other strawberry pests, they are used only to a limited extent and are primarily perceived as options meant for organic agriculture. Earlier field studies demonstrated that alternating or combining biopesticides with chemical pesticides are important in strawberry IPM for *L. hesperus* management [11]. This approach will help reduce the total quantity of chemical pesticides used, takes advantage of alternative control methods, helps reduce resistance from continuous use of chemical pesticides and promotes strategies that utilize a variety of control options. Additionally, using combinations of chemical and biological pesticides improves the efficacy through synergistic or additive actions [12,13,14,15,16]. Chemical or botanical pesticides at regular or lower label rates weaken the pests and improve infection by entomopathogenic fungi, such as *Beauveria bassiana* and *Metarhizium* spp. Rotating chemical and non-chemical alternatives or pesticides with different modes of action reduces the risk of resistance and helps to maintain pest control efficacy. Two field studies were conducted, in 2013 and 2015, to further evaluate the role of non-chemical alternatives in managing arthropod pests in strawberry in combinations and/or rotations.

## 2. Materials and Methods

### 2.1. Twospotted Spider Mite

In 2013, a small plot study was conducted in a conventional strawberry field at Manzanita Berry Farms in Santa Maria, California, to evaluate the efficacy of commonly used chemical miticides along with botanical and microbial pesticides on *T. urticae* and predatory mites (*Phytoseiulus persimilis* and *Neoseiulus californicus*) that are either naturally occurring or those remaining from an earlier release (more than 4–5 weeks prior to the study) for controlling pest mites. The following 10 treatments were evaluated in the study: (i) Untreated control, (ii) Acramite 50 WS (bifenazate) at 1.12 kg/ha, (iii) Agri-Mek SC (abamectin) at 313.5 mL/ha), (iv) BotaniGard ES (entomopathogenic fungus *B. bassiana*) at a lower label rate of 2.3 L/ha + Acramite 50 WS at the lowest label rate of 0.84 kg/ha, (v) Eco-Mite (rosemary + cotton seed oils) at 1% concentration, (vi) Fujimite 5 EC (fenpyroximate) at 2.3 L/ha, (vii) Fujimite XLO (fenpyroximate) at 2.3 L/ha, (viii) Grandevo (*Chromobacterium subtsugae* strain PRAA4-1) at 2.2 kg/ha, (ix) Venerate XC (*Burkholderia rinojensis* strain A396) at 18.7 L/ha and (x) Nealta SC (cyflumetofen) at 1 L/ha. Spray solutions were applied at 1403 L/ha rate using Induce, a non-ionic surfactant, at 0.25% vol/vol for all treatments except for the treatment with *B. bassiana*, where Kinetic, an organo-silicon surfactant, was used. Each treatment consisted of a 4.6 m long and 1.7 m wide (four rows of plants/bed) strawberry plot within a bed and treatments were replicated four times (i.e., four beds) in a randomized complete block design. Treatments were applied early in the morning on 16 and 23 May 2013 using a CO_2_ pressurized backpack sprayer that has six hollow cone nozzles covering the entire width of the bed. The number of eggs and mobile stages of *T. urticae* and predatory mites (*Phytoseiulus persimilus* and *Neoseiulus* spp.) were counted 3 and 7 days after each application from the sampled leaflets. On each of these sampling dates, 10 mid-tier leaflets were collected from 10 random plants in the middle 3.6 m of each plot. Each leaflet was passed through a mite brushing machine (Leedom Enterprises, Mi-Wuk Village, CA, USA) five times and eggs and mobile stages of mites collected on a rotating glass disc of the machine were counted under a microscope. Data were analyzed using ANOVA and significant means were separated using Tukey’s HSD test. Percentage change data in *T. urticae* after two spray applications relative to untreated control were subjected to logarithmic transformation before analysis. Due to a technical error, pre-treatment data were lost and treatment efficacy could be compared only for post-treatment counts. 

### 2.2. Western Tarnished Plant Bug and Other Insect Pests

In 2015, a large plot study was conducted in a conventional strawberry field at Sundance Berry Farms in the Santa Maria area in California. The following treatment regimens were used in different combinations and rotations and evaluated for their efficacy against insect pests with a particular emphasis on *L. hesperus* (Table 1).

**Chemical pesticides:** Six chemical pesticides from different Insecticide Resistance Action Committee (IRAC) mode of action group were used.

IRAC Group 3A—Pyrethrins: Proprietary formulation and bifenthrin (Brigade)

IRAC Group 4A—Neonicotinoids: Acetamiprid (Assail 70 WP)

IRAC Group 4C—Sulfoximines: Sulfoxaflor (Sequoia)

IRAC Group 4D—Butenolides: Flupyradifurone (Sivanto)

IRAC Group 9C—Flonicamid: Flonicamid (Beleaf 50 SG)

IRAC Group 15—Benzoylureas: Novaluron (Rimon 0.83 EC)

**Botanical pesticide:** Azadirachtin is an insecticide, insect growth regulator, antifeedant and a repellent. Different formulations of azadirachtin used in this study were—AzaGuard (3% azadirachtin), Neemix (4.5% azadirachtin) and Debug Turbo (65.8% of oils margosa and 0.7% azadirachtin). Cold pressed neem (37.5 or 75 ppm) or natural pyrethrum (0.5 or 0.75%) were also present in combination with *B. bassiana* in three products used in the study.

**Entomopathogenic fungi:** Three fungi used in the study include *B. bassiana* with cold pressed neem at 75 ppm (XPULSE), pyrethrum at 0.75% (XPECTRO), or both cold pressed neem at 37.5 ppm and pyrethrum at 0.5% (XCEDE), *Isaria fumosorosea* (Pfr-97 20% WDG) and *Metarhizium brunneum* (Met52 EC).

**Mechanical:** Vacuuming was done with a commercial tractor-mounted bug vacuum twice a week with one pass each time at a speed of 3.2 km/h.

The study included 12 treatments where Assail 70 WP alone and vacuuming alone were grower standards along with an untreated control (Table 1). Treatments were administered on 26 August, 2 and 9 September using a tractor-mounted sprayer at 935 L/ha rate. Each treatment consisted of six 22.9 m long and 1.7 m wide beds (four rows of plants/bed) and four replications distributed in a randomized complete block design. Before the first treatment and 6 days after each treatment, 20 plants from the middle two beds in each plot were randomly sampled for insect pests and natural enemies by gently beating each plant 3–4 times with the lid of a shallow plastic container and counting the number of arthropods dislodged into the container. The number of young and old nymphs and adult *L. hesperus*, adults and nymphs of *F. occidentalis*, adult *T. vaporariorum*, adults or immature stages of natural enemies including big-eyed bugs (*Geocoris* spp.), minute pirate bugs (*Orius* spp.), lacewings (*Chrysopa* spp. and *Hemerobius* spp.), damsel bugs (*Nabis* spp.), ladybeetles (Coccinellidae), parasitic wasps (*Anaphes iole*), predatory thrips (Thripidae), predatory midge larvae (Syrphidae) and spiders were counted from each sample plant. Data were subjected to ANOVA and significant means were separated using Tukey’s HSD test. Percentage change data in *L. hesperus* after three applications relative to pre-treatment counts were subjected to logarithmic transformation before analysis.

## 3. Results

### 3.1. Twospotted Spider Mite

**Figure 1:**
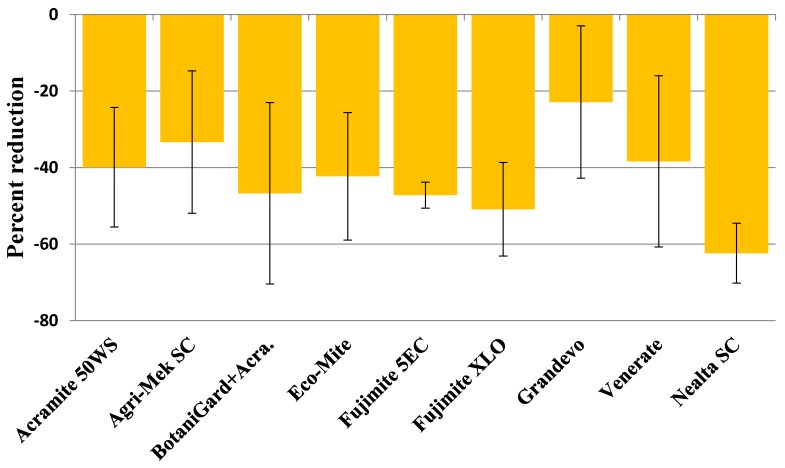
Percent reduction in eggs and mobile stages of *T. urticae* in different treatments relative to untreated control on the last observation date after the second spray application.

Number of eggs and mobile stages of *T. urticae* were higher in untreated control compared to the treated plots throughout the observation period but there were no statistically significant differences (*p* ≥ 0.05) (Table 2). When the percent change in mite populations in treatments relative to those in untreated control was compared after two spray applications, Nealta SC had the highest reduction in egg and mobile stages (62.3%) followed by Fujimite XLO (50.9%) and Fujimite 5 EC (47.2%) (Figure 1). While Acramite 50WS alone resulted in a 39.9% reduction in all life stages of mites, its lower rate combined with BotaniGard ES provided a 46.7% control. Control provided by Eco-Mite (42.2%) and Venerate XC (38.4%) were close to the control from Acramite 50WS, followed by Agri-Mek SC (33.3%) and Grandevo (22.9%). However, there were no statistically significant differences (*p* > 0.05) among these treatments.

Predatory mite populations did not statistically differ among various treatments except on 7 days after the first spray for mobile stages where Fujimite 5 EC had the highest number followed by BotaniGard ES+lower rate of Acramite 50 WS, Venerate XC, Fujimite XLO (*p* = 0.0046, Table 3). In general, plots that were treated with Venerate XC and Nealta SC had a relatively higher number of predatory mites while those treated with Acramite 50 WS and Agri-Mek SC had fewer although differences were not significant (*p* ≥ 0.05). Considering the recent resistance issues with abamectin and bifenazate in *T. urticae* populations (Personal communication with growers and pest control professionals), information on the efficacy of other chemical and non-chemical alternatives against *T. urticae* and safety to predatory mites is very important in developing IPM strategies.

### 3.2. Western Tarnished Plant Bug

**Figure 2:**
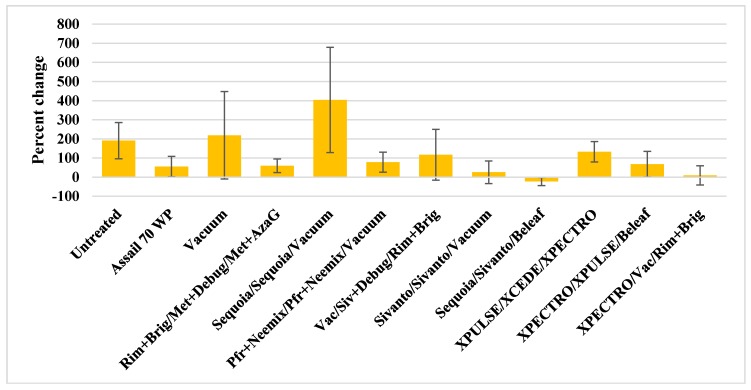
Percent change in nymph and adult *L. hesperus* numbers from pre-treatment to the end of the study.

Populations of *L. hesperus* were very high during the study period (treatment threshold 1 nymph/20 plants) and control was difficult, in general (Table 4). Significant treatment differences (*p* = 0.01) were seen only for older nymphs after the second and third application of treatments. When the percent change in all life stages of *L. hesperus* from pre-treatment to the end of the study were compared, only the Sequoia/Sivanto/Beleaf 50 SG treatment reduced pest numbers by 22.4% (Figure 2). Rest of the treatments varied in their ability to limit the increase of *L. hesperus* with a minimal increase of 9.5% in XPECTRO/Vacuum/Rimon 0.83 EC+Brigade treatment followed by a 25.5% increase in Sivanto/Sivanto/Vacuum treatment. There was a 54.9% increase in *L. hesperus* in the chemical standard Assail 70 WP and 218.8% increase in the vacuumed plots compared to 190.8% increase in the untreated control. Except for the XPECTRO/XCEDE/XPECTRO treatment that saw a 132.5% increase, the remaining treatments that had one or more entomopathogenic fungi limited the increase between 60–79%.

Treatments were not statistically different (*p* ≥ 0.05) for *F. occidentalis*, *T. vaporariorum*, or natural enemy populations (Table 5). Number of *F. occidentalis* increased in all treatments post-treatment except for a decrease in Rimon 0.83 EC+Brigade/Met52 EC+Debug Turbo/Met52 EC+AzaGuard and XPULSE/XCEDE/XPECTRO treatments. In general, *T. vaporariorum* adults occurred at low levels during the sampling period. After three weeks of administering the treatments, a reduction in their numbers was seen in Vacuum/Sivanto+Debug Turbo/Rimon 0.83 EC+Brigade, Sivanto/Sivanto/Vacuum and Rimon 0.83 EX+Brigade/Met52 EC+Debug Turbo/Met52 EC+AzaGuard treatments. Natural enemy populations were relatively higher in Pfr-97 20% WDG+Neemix/Pfr-97 20% WDG+Neemix/Vacuum followed by Sequoia/Sivanto/Beleaf 50 SG and Sequoia/Sequoia/Vacuum and XPULSE/XCEDE/XPECTRO when the pre- and post-treatment counts were compared (Table 5).

## 4. Discussion

These results show that both chemical and non-chemical alternatives vary in their efficacy depending on the combinations and rotation. There is a general perception that chemical pesticides are more efficacious and consistent in their control compared to non-chemical alternatives but it was not the case in this study. Similar studies in California strawberries showed variation in the efficacy of various products in different years probably due to a number of factors including seasonal fluctuations in pest populations, resistant pest populations in different fields and inherent challenges in conducting large field studies [11,13]. While the 2013 study used small plots typical for miticide evaluation experiments, the 2015 study used fairly large plots as *L. hesperus* is very mobile. Although all reasonable efforts were made to choose fields with uniform pest populations, natural variation within each field could have partly contributed to a lack of statistically significant differences. In spite of these inherent variations and lack of significant differences in some instances in field studies such as ours, strawberry growers and pest control professionals still find numerical differences useful in making pest management decisions (Personal communication with various growers and pest control professionals). These data also help in deciding treatment strategies for follow up studies.

To reduce the risk of insecticide resistance development, and to maintain pest control efficacy, effective chemicals from different modes of action groups, and non-chemical alternatives such as botanical and microbial pesticides, should be used in combinations and rotations [16,17,18,19]. Combinations are more effective through synergistic or additive activity in some cases, while rotations are more effective in other cases. Since some biopesticides are very expensive compared to chemical pesticides, their use is limited to certain situations. However, biopesticides can play a critical role in reducing insecticide resistance and offer significant long-term benefits. Some recent studies have shown additional benefits of entomopathogenic fungi, other than pest management, as they can also promote crop growth, health and antagonize plant pathogens [20,21,22,23]. These new roles can offer additional benefits and offset the higher costs associated with entomopathogenic fungi-based biopesticides.

While predatory mite releases are commonly practiced in California strawberries for spider mite control, using bug vacuums on some farms is the primary non-chemical alternative practice for managing *L. hesperus*. Similar to the findings of Pickel et al. [5] or Joseph and Bolda [6], vacuuming was not able to control *L. hesperus* populations when used alone or in some rotation treatments in our study. There is a need to integrate a variety of pest management practices both to improve the control efficacy against pests such as *L. hesperus* as well as to promote IPM practices. Other strawberry studies showed combining and rotating chemical pesticides with botanical and microbial pesticides is an effective strategy for managing *L. hesperus* in strawberry [11]. Although the combinations or rotations of entomopathogenic fungi with other control options did not result in statistically significant reduction in *L. hesperus* numbers, in general, *L. hesperus* population increase was somewhat limited in these treatments compared to the untreated control and where bug vacuum alone or with chemical pesticides were used. Such an improved efficacy of entomopathogenic fungi with chemical pesticides was also observed in a number of other studies. For example, the combination of *B. bassiana* and azadirachtin was more effective in controlling the rice root aphid, *Rhopalosiphum rufiabdominale* and the honeysuckle aphid, *Hyadaphis foeniculi*, in organic celery [14]. Prolonged and improved control of the citrus red mite, *Panonychus citri,* was seen when *B. bassiana* was applied with a lower rate of pyridaben [24]. Similarly, pest control efficacy improved when *B. bassiana* and *M, anisopliae* were used with insect growth regulators [12,25]. Understanding the efficacy of individual treatment choices as well as combination and rotation strategies helps growers make appropriate decisions to manage pest populations.

## 5. Conclusions

These studies demonstrate the efficacy of a variety of control options for managing major arthropod pests in strawberry, their impact on natural enemy populations, and present various ideas for developing IPM strategies. The 2015 study was probably the first large scale field study for *L. hesperus* where almost all available chemical and non-chemical control strategies were evaluated and identified potential options. An effective IPM strategy always employs multiple control options and pesticides from different mode of action groups as needed.

## Figures and Tables

**Table 1:** List of treatments and application rates used for managing *L. hesperus* and other insect pests.

Treatment	1st Application (Rate/ha)	2nd Application (Rate/ha)	3rd Application (Rate/ha)
**1**	Untreated	Untreated	Untreated
**2**	Assail 70 WP (210 g)	Assail 70 WP (210 g)	Assail 70 WP (210 g)
**3**	Vacuum	Vacuum	Vacuum
**4**	Rimon 0.83 EC (877 mL) + Brigade (1.1 kg)	Met52 EC (16 fl oz) + Debug Turbo (7.6 L)	Met52 EC (1.2 L) + AzaGuard (1.2 L)
**5**	Sequoia (329 mL)	Sequoia (329 mL)	Vacuum
**6**	Pfr-97 20% WDG (2.2 kg) + Neemix (658 mL)	Pfr-97 20% WDG (2.2 kg) + Neemix (658 mL)	Vacuum
**7**	Vacuum	Sivanto (1 L) + Debug Turbo (7.6 L)	Rimon 0.83 EC (877 mL) + Brigade (1.1 kg)
**8**	Sivanto (1 L)	Sivanto (1 L)	Vacuum
**9**	Sequoia (329 mL)	Sivanto (1 L)	Beleaf 50 SG (196 g)
**10**	XPULSE (2.3 L)	XCEDE (2.3 L)	XPECTRO (2.3 L)
**11**	XPECTRO (2.3 L)	XPULSE (2.3 L)	Beleaf 50 SG (196 g)
**12**	XPECTRO (2.3 L)	Vacuum	Rimon 0.83 EC (877 mL) + Brigade (1.1 kg)

**Table 2:** Mean number and standard error of eggs and mobile stages (nymphal and adult) of *T. urticae* per leaflet 3 and 7 days after each treatment (DAT).

Treatment	I Spray 3DAT		I Spray 7DAT		II Spray 3DAT		II Spray 7DAT	
	Egg	Mobile	Egg	Mobile	Egg	Mobile	Egg	Mobile
Untreated	109.05 ± 2.73	23.25 ± 1.72	111.60 ± 15.66	20.40 ± 0.52	133.00 ± 9.61	22.20 ± 1.91	85.00 ± 5.97	23.8 ± 1.51
Acramite 50WS	87.60 ± 25.16	15.6 ± 7.42	75.80 ± 23.08	7.80 ± 2.22	94.00 ± 33.76	14.00 ± 1.97	57.80 ± 18.24	10.80 ± 3.20
Agri-Mek SC	72.45 ± 15.26	10.80 ± 1.78	69.20 ± 17.49	12.80 ± 3.21	84.20 ± 15.31	19.40 ± 3.58	56.40 ± 19.50	19.60 ± 3.99
BotaniGard ES+Acramite 50WS	90.90 ± 17.67	12.15 ± 2.08	79.00 ± 14.81	8.20 ± 1.14	86.40 ± 15.31	15.80 ± 2.49	32.40 ± 6.74	20.40 ± 12.45
Eco-Mite	42.45 ± 15.53	5.25 ± 2.59	66.80 ± 4.99	11.40 ± 3.01	69.00 ± 7.15	10.80 ± 2.13	48.20 ± 19.36	14.00 ± 5.25
Fujimite 5 EC	65.10 ± 19.32	8.85 ± 2.79	68.80 ± 14.77	8.00 ± 1.34	62.80 ± 3.90	13.40 ± 3.15	45.80 ± 6.42	12.20 ± 1.28
Fujimite XLO	71.25 ± 18.93	19.50 ± 8.78	85.00 ± 28.52	11.60 ± 1.20	91.20 ± 15.40	15.00 ± 4.52	43.40 ± 15.78	10.40 ± 1.08
Grandevo	75.60 ± 10.22	14.40 ± 2.86	85.80 ± 28.15	12.40 ± 2.58	111.20 ± 15.87	14.80 ± 3.78	64.60 ± 15.69	16.20 ± 4.36
Venerate XC	45.60 ± 10.77	16.35 ± 2.86	54.20 ± 18.15	16.00 ± 5.65	65.60 ± 25.57	12.80 ± 5.66	48.20 ± 14.52	14.60 ± 5.19
Nealta SC	66.00 ± 22.99	18.30 ± 9.76	51.00 ± 11.34	8.80 ± 2.4	82.60 ± 8.95	8.40 ± 2.02	31.40 ± 7.46	10.40 ± 3.33
***p* value**	0.2310	0.1702	0.4023	0.0490	0.1627	0.2441	0.2049	0.5918

**Table 3:** Mean number and standard error of eggs and mobile stages (nymphal and adult) of predatory mites per leaflet 3 and 7 days after each treatment (DAT).

	I Spray 3DAT		I Spray 7DAT		II Spray 3DAT		II Spray 7DAT	
	Egg	Mobile	Egg	Mobile	Egg	Mobile	Egg	Mobile
Untreated	0.00	1.65 ± 1.05	1.00 ± 0.38	0.60 ± 0.20 b *	1.80 ± 0.76	2.20 ± 0.68	2.60 ± 1.00	4.20 ± 1.64
Acramite 50WS	0.45 ± 0.28	0.60 ± 0.24	1.00 ± 0.60	0.60 ± 0.38 b	1.00 ± 0.50	1.20 ± 0.40	1.00 ± 0.38	1.20 ± 0.51
Agri-Mek SC	1.20 ± 0.24	0.45 ± 0.28	1.00 ± 0.60	0.20 ± 0.20 b	0.60 ± 0.20	1.40 ± 0.68	0.40 ± 0.23	2.60 ± 1.00
BotaniGard ES+Acramite 50WS	0.45 ± 0.28	0.60 ± 0.34	0.80 ± 0.46	1.60 ± 0.92 ab	2.00 ± 1.20	2.60 ± 2.34	1.60 ± 0.65	4.20 ± 2.29
Eco-Mite	0.30 ± 0.17	0.75 ± 0.56	2.20 ± 0.88	0.60 ± 0.60 b	0.80 ± 0.46	1.60 ± 0.56	2.00 ± 0.40	3.00 ± 0.88
Fujimite 5 EC	0.60 ± 0.34	0.45 ± 0.28	1.60 ± 0.86	4.00 ± 1.34 a	1.00 ± 0.75	1.00 ± 0.20	2.00 ± 0.76	2.60 ± 1.54
Fujimite XLO	0.90 ± 0.38	0.60 ± 0.24	1.60 ± 0.65	1.20 ± 0.23 ab	1.40 ± 0.50	0.60 ± 0.38	3.00 ± 1.24	2.60 ± 1.36
Grandevo	0.45 ± 0.28	0.00	0.60 ± 0.38	0.80 ± 0.32 b	1.80 ± 0.75	0.60 ± 0.38	1.80 ± 0.68	2.40 ± 0.56
Venerate XC	2.40 ± 1.22	1.65 ± 1.26	1.80 ± 0.82	1.40 ± 0.50 ab	1.40 ± 0.88	2.80 ± 1.20	2.00 ± 0.23	5.20 ± 1.51
Nealta SC	0.90 ± 0.38	1.35 ± 0.56	1.80 ± 0.68	0.40 ± 0.23 b	1.60 ± 0.80	3.60 ± 1.74	2.00 ± 0.51	3.80 ± 0.82
***p* value**	0.0570	0.3899	0.7879	0.0046	0.8894	0.5535	0.3962	0.6581

* Means followed by the same or no letter within each column are not statistically significant, Tukey’s HSD.

**Table 4:** Number of *L. hesperus* nymphs (young and old) and adults per 20 plants before and after each treatment.

Treatment	Pretreatment				Post I Spray				Post II Spray				Post III Spray			
	1–3 Instar	4–5 Instar	Adult	All Stages	1–3 Instar	4–5 Instar	Adult	All Stages	1–3 Instar	4–5 Instar	Adult	All Stages	1–3 Instar	4–5 Instar	Adult	All Stages
Untreated	18.50 ± 6.70	2.50 ± 1.32	2.00 ± 0.57	23.00 ± 8.25	25.50 ± 5.08	5.75 ± 2.80	2.50 ± 1.50	33.75 ± 8.37	24.00 ± 6.82	6.75 ± 2.39 a *	6.75 ± 1.37	37.50 ± 10.51	33.50 ± 14.38	6.25 ± 1.11 a	12.00 ± 3.89	51.75 ± 18.25
Assail 70 WP	20.75 ± 8.09	3.75 ± 2.17	2.50 ± 1.55	27.00 ± 11.27	21.00 ± 4.77	4.00 ± 2.48	1.50 ± 0.95	26.50 ± 7.35	22.00 ± 8.12	5.50 ± 1.85 ab	4.75 ± 1.31	32.25 ± 10.34	20.00 ± 9.37	2.25 ± 0.63 ab	6.50 ± 3.66	28.75 ± 12.75
Vacuum	21.25 ± 7.39	2.50 ± 0.64	2.25 ± 1.10	26.00 ± 8.45	15.50 ± 2.53	3.75 ± 1.18	1.50 ± 0.95	20.75 ± 3.27	22.75 ± 6.96	6.25 ± 2.65 ab	4.25 ± 1.49	33.25 ± 10.72	25.25 ± 7.34	3.25 ± 1.25 ab	4.50 ± 1.19	33.00 ± 9.60
Rim+Brig/Met+Debug/Met+AzaG	20.75 ± 5.50	2.50 ± 1.32	1.25 ± 0.94	24.50 ± 6.66	14.25 ± 5.15	2.50 ± 0.86	2.25 ± 1.10	19.00 ± 6.41	16.25 ± 5.53	1.25 ± 0.25 b	5.25 ± 1.75	22.75 ± 7.38	30.25 ± 6.83	1.00 ± 0.70 b	6.25 ± 2.53	37.50 ± 8.70
Sequoia/Sequoia/Vacuum	11.50 ± 4.57	1.75 ± 0.85	0.75 ± 0.48	14.00 ± 5.80	11.50 ± 2.72	2.25 ± 1.31	3.25 ± 1.25	17.00 ± 4.81	13.25 ± 4.95	2.75 ± 1.11 ab	4.00 ± 1.78	20.00 ± 6.99	26.50 ± 10.63	0.50 ± 0.28 b	7.75 ± 3.12	34.7513.36
Pfr+Neemix/Pfr+Neemix/Vacuum	21.25 ± 3.92	2.00 ± 0.91	1.50 ± 0.95	24.75 ± 4.02	19.75 ± 5.36	4.75 ± 2.75	1.75 ± 0.75	26.25 ± 6.77	21.00 ± 6.82	3.50 ± 1.44 ab	6.00 ± 2.16	30.50 ± 9.57	32.50 ± 10.61	2.00 ± 1.41 ab	7.25 ± 2.53	41.75 ± 12.21
Vac?Siv+Debug/Rim+Brig	17.25 ± 8.38	1.00 ± 0.41	1.00 ± 0.71	19.25 ± 8.87	21.00 ± 8.89	3.25 ± 1.43	2.50 ± 1.50	26.75 ± 11.76	18.75 ± 4.25	3.25 ± 1.49 ab	2.50 ± 0.95	24.50 ± 6.38	15.25 ± 6.07	1.75 ± 1.18 b	5.25 ± 2.56	22.25 ± 9.05
Sivanto/Sivanto/Vacuum	30.00 ± 10.97	2.00 ± 0.70	1.75 ± 0.85	33.75 ± 11.79	23.75 ± 5.90	4.00 ± 1.41	2.75 ± 0.47	30.50 ± 6.93	16.75 ± 5.43	6.25 ± 1.49 ab	5.25 ± 1.75	28.25 ± 8.07	21.50 ± 6.18	3.50 ± 1.50 ab	5.25 ± 1.88	30.25 ± 8.83
Sequoia/Sivanto/Beleaf	31.50 ± 12.54	3.50 ± 2.02	0.75 ± 0.25	35.75 ± 14.34	12.50 ± 5.81	4.50 ± 1.19	1.75 ± 0.47	18.75 ± 7.39	23.25 ± 7.87	4.75 ± 1.49 ab	7.50 ± 1.85	35.50 ± 7.22	15.25 ± 3.70	1.50 ± 0.86 b	5.25 ± 1.31	22.00 ± 5.43
XPULSE/XCEDE/XPECTRO	15.00 ± 3.13	1.25 ± 0.94	1.25 ± 0.48	17.50 ± 4.33	22.00 ± 4.96	4.00 ± 0.70	2.25 ± 0.85	28.25 ± 5.31	24.00 ± 8.91	5.00 ± 1.47 ab	3.00 ± 0.81	32.00 ± 9.83	29.00 ± 6.85	2.25 ± 0.75 ab	6.00 ± 2.80	37.25 ± 9.93
XPECTRO/XPULSE/Beleaf	17.00 ± 6.01	1.50 ± 0.64	0.75 ± 0.75	19.25 ± 6.96	20.75 ± 6.70	3.75 ± 1.37	1.75 ± 0.47	26.25 ± 7.60	24.50 ± 3.38	5.75 ± 2.05 ab	5.50 ± 1.70	35.75 ± 5.93	16.25 ± 5.12	2.00 ± 1.08 ab	4.50 ± 2.32	22.75 ± 8.19
XPECTRO/Vac/Rim+Brig	32.50 ± 8.51	3.50 ± 1.44	1.50 ± 0.86	37.50 ± 9.34	31.00 ± 6.21	7.00 ± 2.64	4.00 ± 1.29	42.00 ± 9.12	26.00 ± 9.89	7.25 ± 1.54 a	7.00 ± 1.58	40.25 ± 12.57	18.00 ± 6.25	3.00 ± 1.47 ab	9.25 ± 3.32	30.25 ± 10.53
***p* value**	0.7107	0.8608	0.8794	0.7562	0.1077	0.8097	0.7849	0.1493	0.6448	0.0090	0.2466	0.2422	0.3936	0.0074	0.4233	0.4619

* Means followed by the same or no letter are not significantly different (Tukey’s HSD).

**Table 5:** Number of western flower thrips (*F. occidentalis*), greenhouse whiteflies (*T. vaporariorum*) and various natural enemies before and after each treatment.

Treatment	Western Flower Thrips					Greenhouse Whiteflies				Natural Enemy Complex		
	Pre-Treat.	Post I Treat.	Post II Treat.	Post III Treat.	Pre-Treat.	Post I Treat.	Post II Treat.	Post III Treat.	Pre-Treat.	Post I Treat.	Post II Treat.	Post III Treat.
Untreated	7.00 ± 3.00	15.75 ± 5.66	13.25 ± 4.21	12.25 ± 2.28	0.25 ± 0.25	1.00 ± 0.41	0.25 ± 0.25	1.75 ± 0.85	1.25 ± 0.63	2.25 ± 0.85	4.75 ± 3.54	2.25 ± 1.10
Assail 70 WP	9.00 ± 1.82	21.00 ± 10.32	13.25 ± 5.87	13.50 ± 2.60	0.25 ± 0.25	0.25 ± 0.25	1.25 ± 1.25	0.50 ± 0.50	2.25 ± 1.93	5.25 ± 4.60	3.75 ± 2.25	2.00 ± 1.41
Vacuum	4.75 ± 1.11	20.50 ± 6.20	15.50 ± 4.66	15.75 ± 4.82	0.75 ± 0.47	1.25 ± 0.94	0.75 ± 0.47	0.75 ± 0.47	2.00 ± 0.91	3.00 ± 1.78	3.00 ± 1.47	1.00 ± 0.57
Rim+Brig/Met+Debug/Met+AzaG	18.25 ± 7.69	12.25 ± 2.86	8.25 ± 2.49	11.50 ± 2.02	0.50 ± 0.50	0.25 ± 0.25	0.25 ± 0.25	0.50 ± 0.50	2.00 ± 1.35	3.50 ± 2.25	1.75 ± 0.47	2.00 ± 0.70
Sequoia/Sequoia/Vacuum	5.50 ± 1.04	17.75 ± 4.95	14.75 ± 4.36	18.25 ± 5.57	0.00	0.25 ± 0.25	0.00	0.50 ± 0.50	0.75 ± 0.47	3.00 ± 2.04	3.00 ± 1.73	2.75 ± 1.60
Pfr+Neemix/Pfr+Neemix/Vacuum	13.25 ± 2.49	18.50 ± 4.25	15.25 ± 4.64	13.50 ± 1.19	0.00	1.00 ± 0.70	0.25 ± 0.25	1.00 ± 0.57	0.25 ± 0.25	2.50 ± 1.89	2.25 ± 1.60	2.50 ± 1.19
Vac?Siv+Debug/Rim+Brig	5.50 ± 2.46	14.25 ± 5.13	11.75 ± 3.70	6.25 ± 2.49	0.75 ± 0.75	0.00	0.00	0.00	1.00 ± 0.70	2.25 ± 0.85	2.25 ± 0.85	2.25 ± 1.03
Sivanto/Sivanto/Vacuum	9.75 ± 1.49	12.50 ± 6.58	11.75 ± 4.32	10.50 ± 3.17	0.25 ± 0.25	0.00	0.00	0.250.25	2.75 ± 1.11	3.50 ± 2.53	5.25 ± 3.94	3.00 ± 0.91
Sequoia/Sivanto/Beleaf	7.25 ± 4.62	14.50 ± 3.96	13.75 ± 2.25	16.75 ± 3.94	0.25 ± 0.25	1.00 ± 1.00	0.00	0.25 ± 0.25	1.00 ± 0.41	6.75 ± 3.81	1.25 ± 0.94	5.50 ± 3.57
XPULSE/XCEDE/XPECTRO	16.50 ± 7.76	16.50 ± 4.73	10.25 ± 2.75	11.75 ± 0.85	0.25 ± 0.25	1.25 ± 0.75	0.00	0.25 ± 0.25	0.75 ± 0.25	3.50 ± 1.55	2.00 ± 1.08	3.25 ± 1.79
XPECTRO/XPULSE/Beleaf	6.00 ± 1.91	17.00 ± 6.86	8.75 ± 2.17	10.25 ± 1.70	0.00	1.25 ± 0.94	0.50 ± 0.50	0.75 ± 0.47	2.50 ± 0.95	3.75 ± 2.78	1.75 ± 0.75	2.00 ± 0.41
XPECTRO/Vac/Rim+Brig	9.75 ± 5.48	18.00 ± 4.43	9.00 ± 4.02	14.50 ± 2.10	0.00	0.25 ± 0.25	0.00	1.00 ± 0.70	1.75 ± 0.75	5.75 ± 0.47	4.25 ± 1.88	2.75 ± 1.18
***p* value**	0.2582	0.8744	0.6570	0.3309	0.8162	0.6930	0.6029	0.4671	0.6241	0.7246	0.4279	0.4619

## References

[1] California Department of Food and Agriculture (CDFA) (2018). California Agricultural Statistics Review, 2016–2017. https://www.cdfa.ca.gov/Statistics/PDFs/2016-17AgReport.pdf.

[2] Zalom F.G., Bolda M.P., Dara S.K., Joseph S. (2014). UC IPM Pest Management Guidelines: Strawberry. University of California Statewide Integrated Pest Management Program.

[3] Dara S.K. (2015). Virus decline of strawberry in California and the role of insect vectors and associated viruses. Plant Health Prog..

[4] Vincent C., Lachance P. (1993). Evaluation of a tractor-propelled vacuum device for management of tarnished plant bug (Heteroptera: Miridae) populations in strawberry plantations. Environ. Entomol..

[5] Pickel C., Zalom F.G., Walsh D.B., Welch N.C. (1995). Vacuums provide limited lygus control in strawberries. Calif. Agric..

[6] Joseph S.V., Bolda M. (2018). Evaluating the potential utility of an electrostatic sprayer and a tractor-mounted vacuum machine for *Lygus hesperus* (Hemiptera: Miridae) management in California’s coastal strawberry. Crop Prot..

[7] California Department of Pesticide Regulation (CDPR) (2015). Summary of Pesticide Use Report Data 2013: Indexed by Commodity. http://www.cdpr.ca.gov/docs/pur/pur13rep/comrpt13.pdf.

[8] Çağatay N.S., Menault P., Riga M., Vontas J., Ay R. (2018). Identification and characterization of Abamectin resistance in *Tetranychus urticae* Koch populations from greenhouses in Turkey. Crop Prot..

[9] Parys K.A., Luttrell R.G., Snodgrass G.L., Portilla M.R. (2018). Patterns of tarnished plant bug (Hemiptera: Miridae) resistance to pyrethroid insecticides in the Lower Mississippi Delta for 2008–2015: Linkage to pyrethroid use and cotton insect management. J. Insect Sci..

[10] Xu D., He Y., Zhang Y., Xie W., Wu Q., Wang S. (2018). Status of pesticide resistance and associated mutations in the two-spotted spider mite, *Tetranychus urticae*, in China. Pest. Biochem. Physiol..

[11] Dara S.K. (2016). Managing strawberry pests with chemical pesticides and non-chemical alternatives. Int. J. Fruit Sci..

[12] Bitsadze N., Jaronski S., Khasdan V., Abashidze E., Abashidze M., Latchininsky A., Samadashvili D., Sokhadze I., Rippa M., Ishaaya I. (2013). Joint action of *Beauveria bassiana* and the insect growth regulators diflubenzuron and novaluron, on the migratory locust, *Locusta migratoria*. J. Pest Sci..

[13] Dara S.K., Dara S.R., Dara S.S. (2013). Endophytic colonization and pest management potential of *Beauveria bassiana* in strawberries. J. Berry Res..

[14] Dara S.K. (2015). Root aphids and their management in organic celery. CAPCA Advis..

[15] Tomilova O.G., Kryukov V.Y., Duisembekov B.A., Yaroslavtseva O.N., Tyurin M.V., Kryukova N.A., Skorokhod V., Dubovskiy I.M., Glupov V.V. (2016). Immune-physiological aspects of synergy between avermectins and the entomopathogenic fungus *Metarhizium robertsii* in Colorado potato beetle larvae. J. Invertebr. Pathol..

[16] Tabashnik B.E. (1989). Managing resistance with multiple pesticide tactics: Theory, evidence, and recommendations. J. Econ. Entomol..

[17] Attique M.N.R., Khaliq A., Sayyed A.H. (2006). Could resistance to insecticides in *Plutella xylostella* (Lep., Plutellidae) be overcome by insecticide mixtures?. J. Appl. Entomol..

[18] Cloyd R.A. (2010). Pesticide mixtures and rotations: Are these viable resistance mitigating strategies. Pest Technol..

[19] Sudo M., Takahashi D., Andow D.A., Suzuki Y., Yamanaka T. (2018). Optimal management strategy of insecticide resistance under various insect life histories: Heterogeneous timing of selection and interpatch dispersal. Evol. Appl..

[20] Sasan R.K., Bidochka M.J. (2012). The insect-pathogenic fungus *Metarhizium robertsii* (Clavicipitaceae) is also an endophyte that stimulates plant root development. Am. J. Bot..

[21] Dara S.K. (2013). Entomopathogenic Fungus *Beauveria bassiana* Promotes Strawberry Plant Growth and Health. https://ucanr.edu/blogs/blogcore/postdetail.cfm?postnum=11624.

[22] Dara S.K., Dara S.S.R., Dara S.S. (2017). Impact of entomopathogenic fungi on the growth, development, and health of cabbage growing under water stress. Amer. J. Plant Sci..

[23] Dara S.S.R., Dara S.S., Dara S.K., Anderson T. (2017). Fighting plant pathogenic fungi with entomopathogenic fungi and other biologicals. CAPCA Advis..

[24] Shi W.-B., Feng M.-G. (2006). Field efficacy of application of *Beauveria bassiana* formulation and low rate pyridaben for sustainable control of citrus red mite *Panonychus citri* (Acari: Tetranychidae) in orchards. Biol. Control.

[25] Hiromori H., Nishigaki J. (1998). Joint action of an entomopathogenic fungus (*Metarhizium anisopliae*) with synthetic insecticides against the scarab beetle, *Anomala cuprea* (Coleoptera: Scarabaeidae) larvae. Appl. Entomol. Zool..

